# Supplementary material for: Successive redox-mediated visible-light ferrophotovoltaics
Source: Nat Commun. 2020 Feb 19;11:966. doi: 10.1038/s41467-020-14763-6 (PMC7031293; doi:10.1038/s41467-020-14763-6)
Supplement: Supplementary file 1 — Supplementary Information [file 41467_2020_14763_MOESM1_ESM.pdf]

Supplementary Information

Successive redox-mediated visible-light ferrophotovoltaics

Y. Noguchi et al.

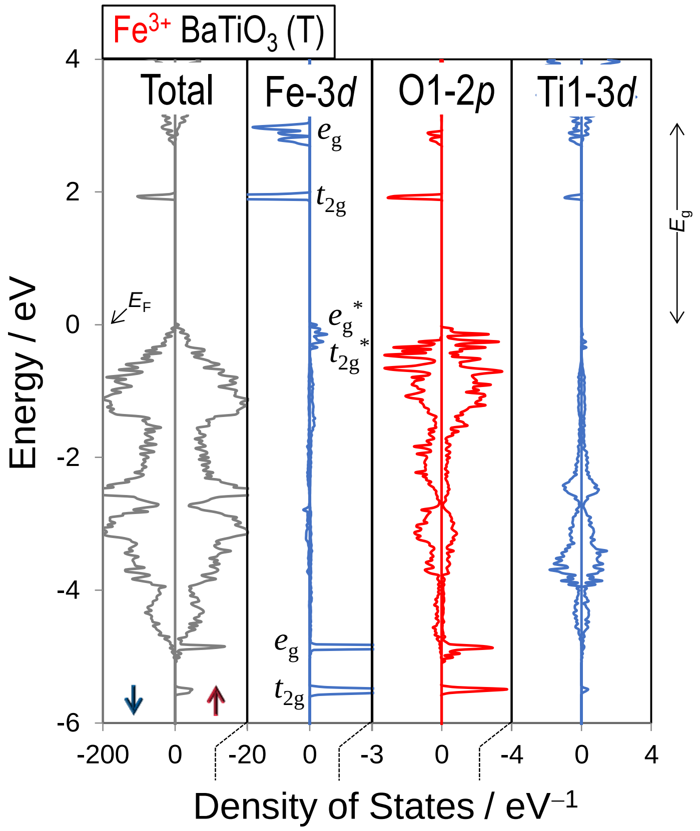

Supplementary Figure 1| Density of states of tetragonal (T) Fe^{3+}-$BaTiO_3$ cell. The results of the $Ba_{27}Ti_{26}FeO_{81}$ (Fe^{3+}) cell (space group *P*4*mm*) over a wide energy range (from –6 eV to 4 eV) are shown. The Fermi level is denoted by E_F. The bandgap is denoted by E_g. The right and left panels indicate the majority (↑) and minority (↓) spin bands, respectively, as indicated in the total the density of states (DOS). As can be seen in the ↑ band, he hybridization between Fe-3*d* and the adjacent orbitals results in the bonding states of t_{2g} (d_{xy}, $d_{xz,}$ and d_{yz}) and e_g (d_{x2-y2} and d_{z2}) in the low-energy range and the antibonding ones of t_{2g}^* and e_g^* in the high-energy range, where asterisk (*) denotes antibonding.

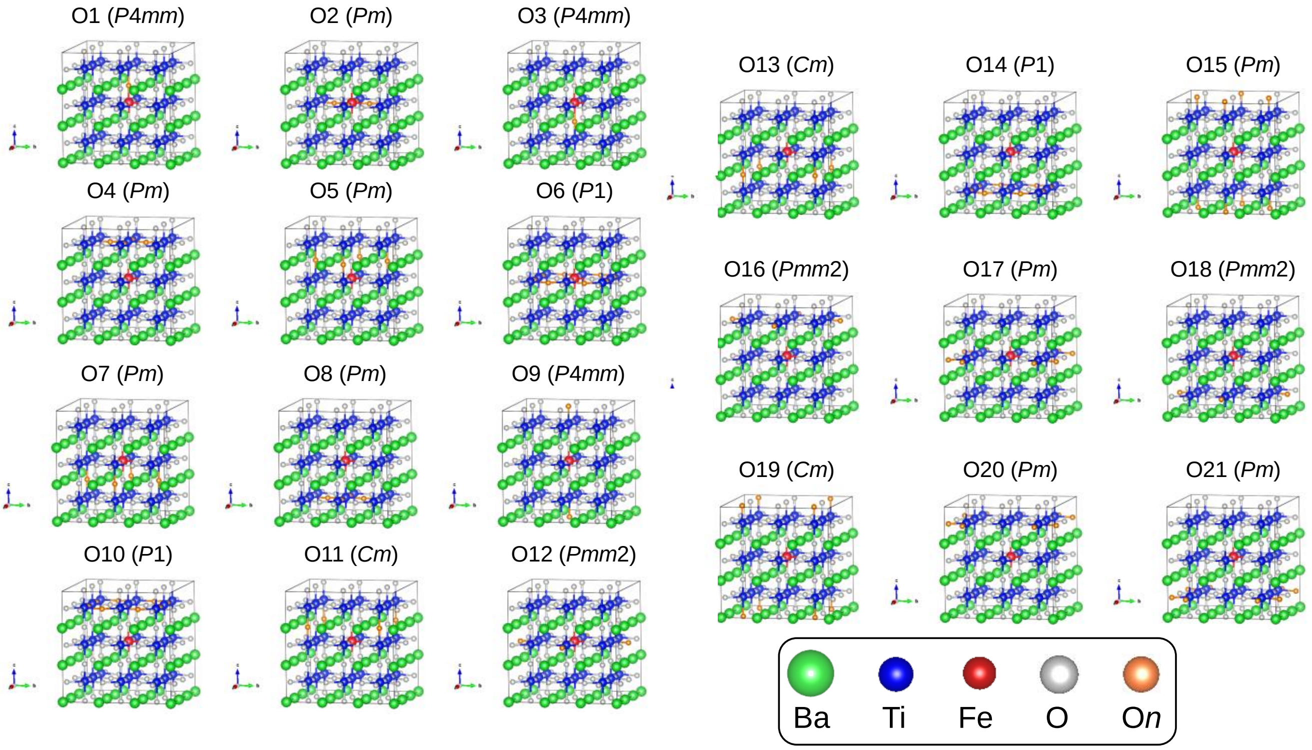

Supplementary Figure 2| Oxygen sites in tetragonal (T) Fe-BaTiO$_3$ cells. In the $Ba_{27}Ti_{26}FeO_{81}$ structure (the supercell of 3 × 3 × 3 created from the optimized $BaTiO_3$ cell with tetragonal *P*4*mm* symmetry), twenty-one types of oxygen atoms (orange) with different site symmetries exist. The symmetry in round brackets denotes the space group after the corresponding (one) oxygen atom is removed in *P*1 symmetry, i.e., an oxygen vacancy ($V_O^{\bullet\bullet}$) is placed on its site.

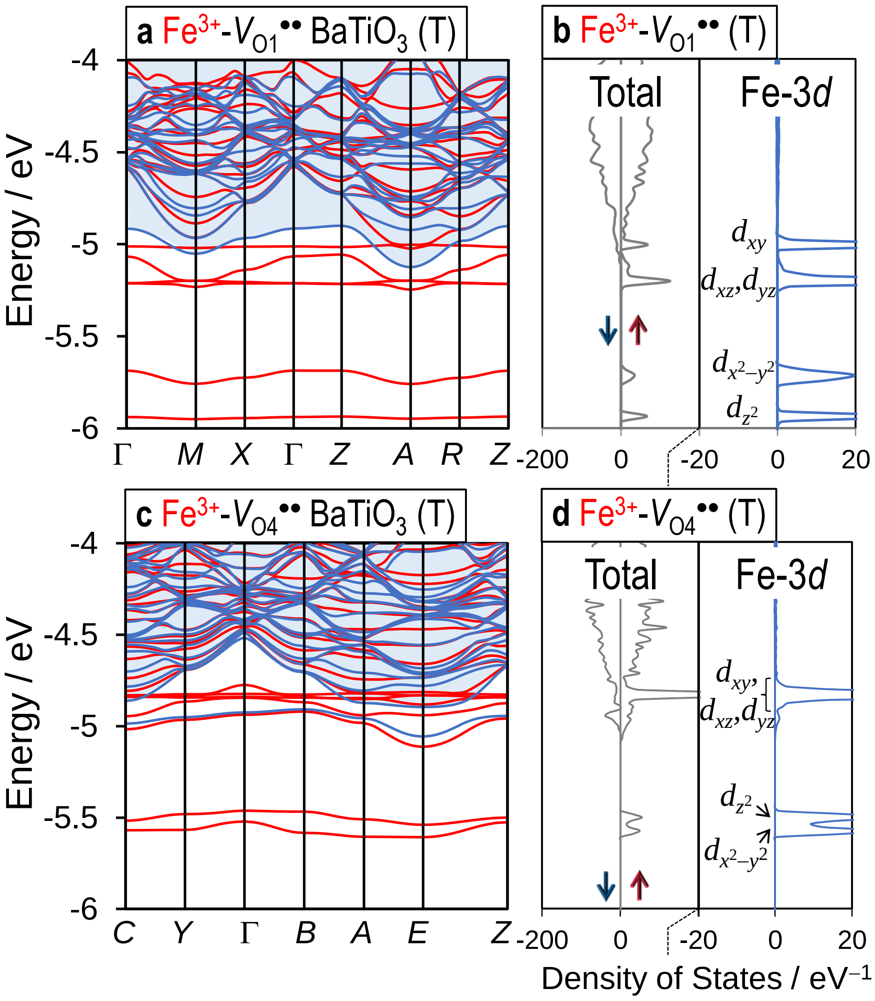

Supplementary Figure 3| Electronic structures of tetragonal (T) Fe^{3+}-$BaTiO_3$ cells with $V_{O1}^{••}$ and $V_{O4}^{••}$. The results in a low energy range from –6 eV to –4 eV of the **a, b** $Ba_{27}Ti_{26}FeO_{80}$ (Fe^{3+}-$V_{O1}^{••}$, space group *P*4*mm*) and **c, d** $Ba_{27}Ti_{26}FeO_{80}$ (Fe^{3+}-$V_{O4}^{••}$, space group *Pm*) cells are shown, where $V_{O1}^{••}$ and $V_{O4}^{••}$ denote the oxygen vacancies on the O1 and O4 sites, respectively. In the band structure, the red and blue lines indicate the majority (↑) and minority (↓) spin components, respectively. The valence band and the conduction band of the host $BaTiO_3$ lattice are coloured light blue and light orange, respectively. Red and blue arrows inside the bandgap denote electron occupying in their corresponding spin bands. In the density of states (DOS), the right and left panels indicate the ↑ and ↓ spin bands, respectively, as indicated in the total DOS. Both the cells exhibit the Fe-3*d* derived bonding (↑) bands of the e_g (d_{x2-y2} and d_{z2}) and t_{2g} (d_{xy}, d_{xz} and d_{yz}) states. All these bands of the Fe^{3+}-$V_{O1}^{••}$ cell are lower by ≈ 0.5 eV than those of the Fe^{3+}-$V_{O4}^{••}$ cell. The lower-lying Fe-3*d* bands **a** originating mainly from a weak ligand field caused by $V_{O1}^{••}$ (adjacent to Fe^{3+}) contributes to a stabilization of the Fe^{3+}-$V_{O1}^{••}$ cell.

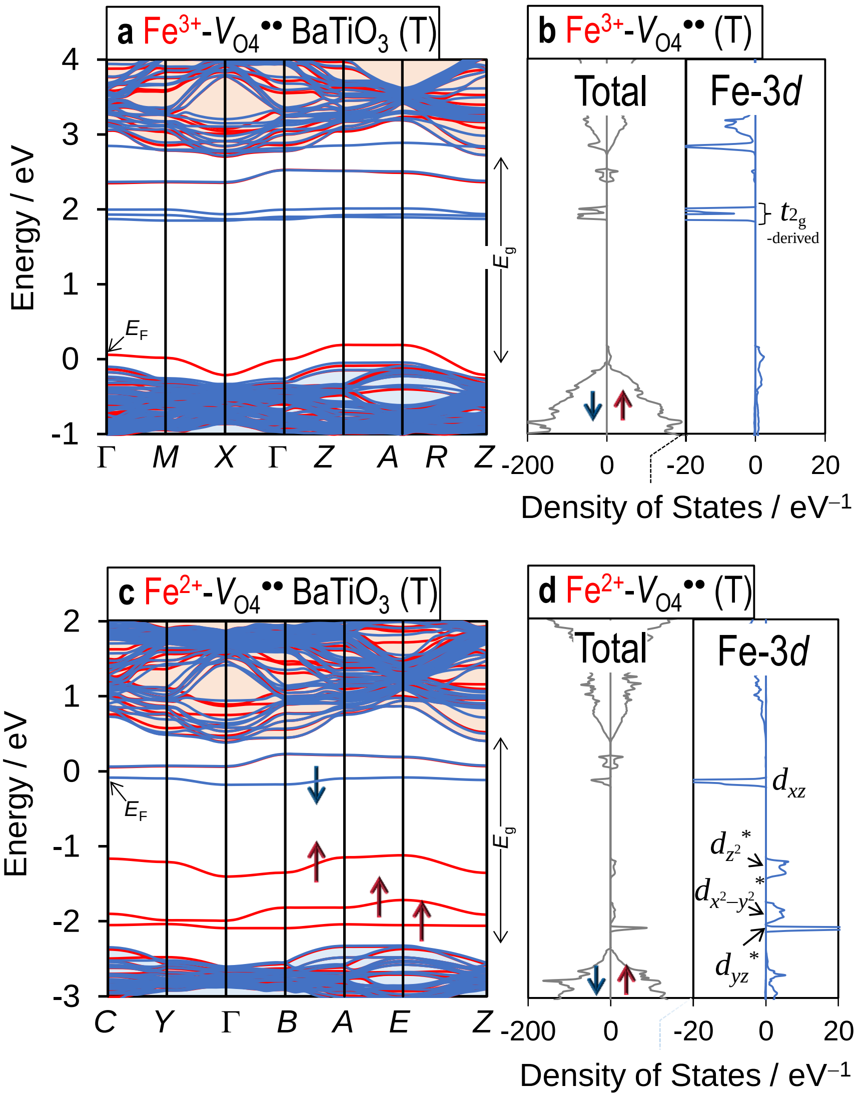

Supplementary Figure 4| Electronic structures of tetragonal (T) Fe-BaTiO₃ cells with $V_{O4}^{••}$. The electronic structures of the **a, b** $Ba_{27}Ti_{26}FeO_{80}$ (Fe^{3+}-$V_{O4}^{••}$, space group *Pm*) and **c, d** $Ba_{27}Ti_{26}FeO_{80}$ (Fe^{2+}-$V_{O4}^{••}$, space group *Pm*) cells are shown, where $V_{O4}^{••}$ denotes the oxygen vacancy on the O4 site. The Fermi level is denoted by E_F. The bandgap is denoted by E_g. The Fe^{3+}-$V_{O4}^{••}$ cell has the empty gap states of t_{2g} (↓) at a depth of ≈ 2.0 eV from the valence band maximum (VBM). Its overall character is similar to that of the Fe^{3+}-$V_{O1}^{••}$ cell (Fig. 4**c, d**). In contrast, the Fe^{2+}-$V_{O4}^{••}$ cell exhibits gap-state features that are different from the Fe^{2+}-$V_{O1}^{••}$ cell (Fig. 4**e, f**), e.g., the d_{xz} (↓) state provides the E_F. Moreover, the Fe^{2+}-$V_{O4}^{••}$ cell has not only the d_{x2-y2}^{*} (↑) state but also the d_{yz}^{*} (↑) and d_{z2}^{*} (↑) states below E_F, which is attributed to its destabilization. The states of, e.g., d_{yz} and d_{yz}^{*} indicate the Fe-3d_{yz} derived bonding and antibonding states, respectively.

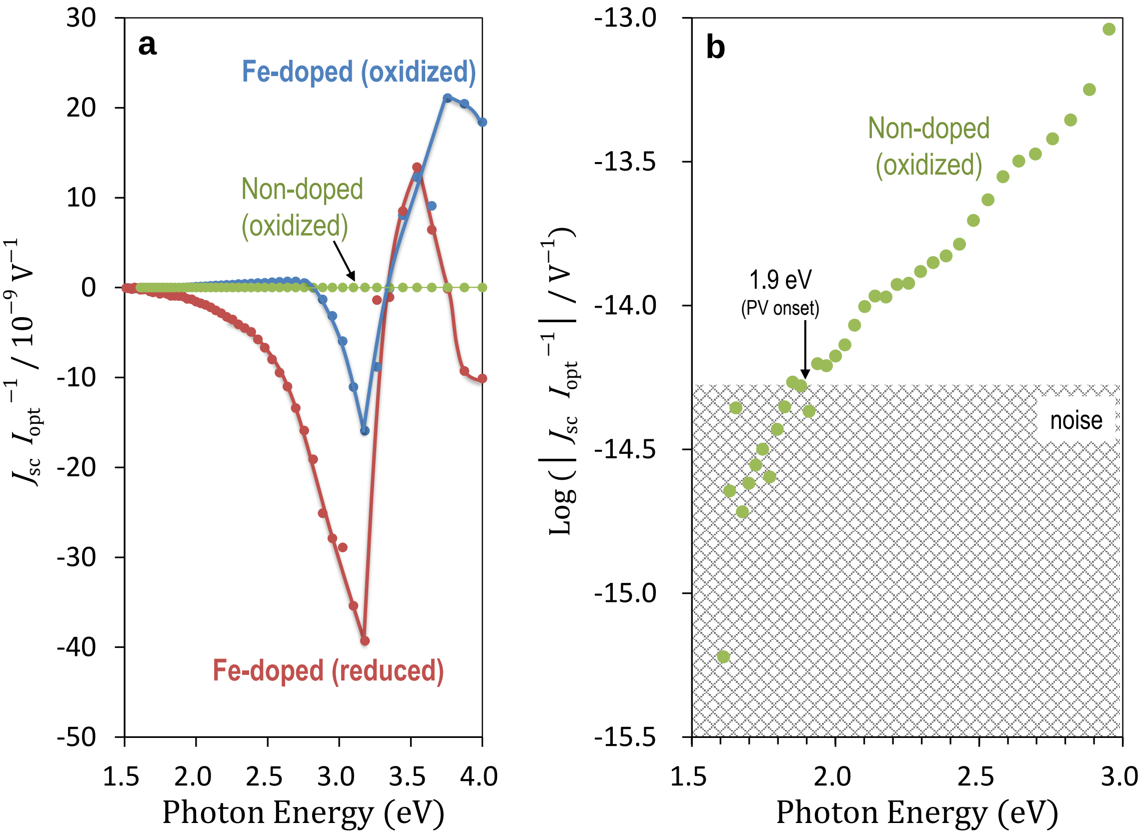

Supplementary Figure 5 Photovoltaic currents *versus* photon energy. The short-circuit current density (J_{sc}) normalized by optical intensity (I_{opt}) is potted as a function of photon energy ($h\nu$) for **a** the reduced and oxidized Fe-doped samples (the same data are shown in Fig. 6**a**, **c**) along with the non-doped sample and **b** the expanded data of the non-doped one. The non-doped sample exhibits a small PV response over the whole $h\nu$ range but indicates an apparent visible-light PV response with an onset at ≈ 1.9 eV. This onset energy accords with that of the Fe-doped sample (oxidized), because unintentional impurity of Fe^{3+} is present with a concentration of several tens ppm even in the non-doped sample (as is in non-doped $BaTiO_3$ ceramics[2,3]).

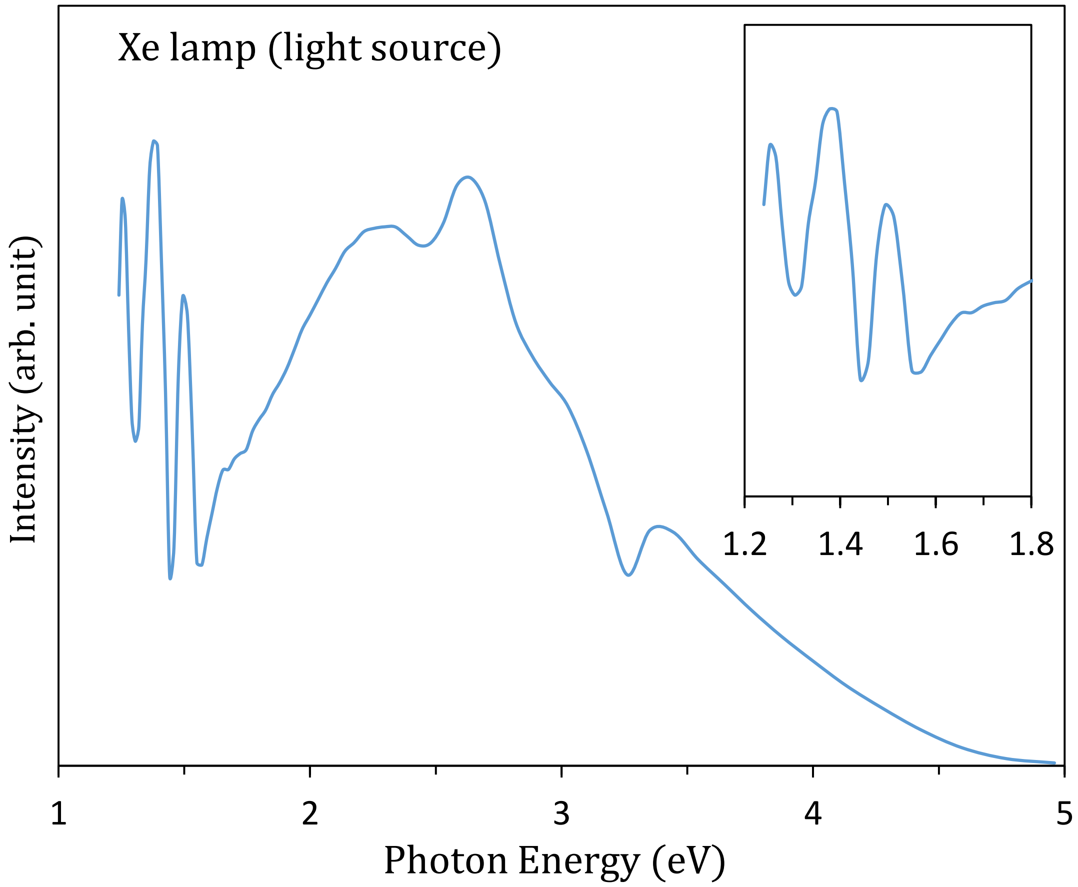

Supplementary Figure 6| Spectrum of xenon lamp used as a light source. The spectral data were collected in the same condition for the PV measurements (Fig. 6 and Supplementary Fig. 5) The light source has the strong emissions in the near-infrared range below 1.55 eV. If the PV onset is below this energy, we cannot determine its energy unambiguously.

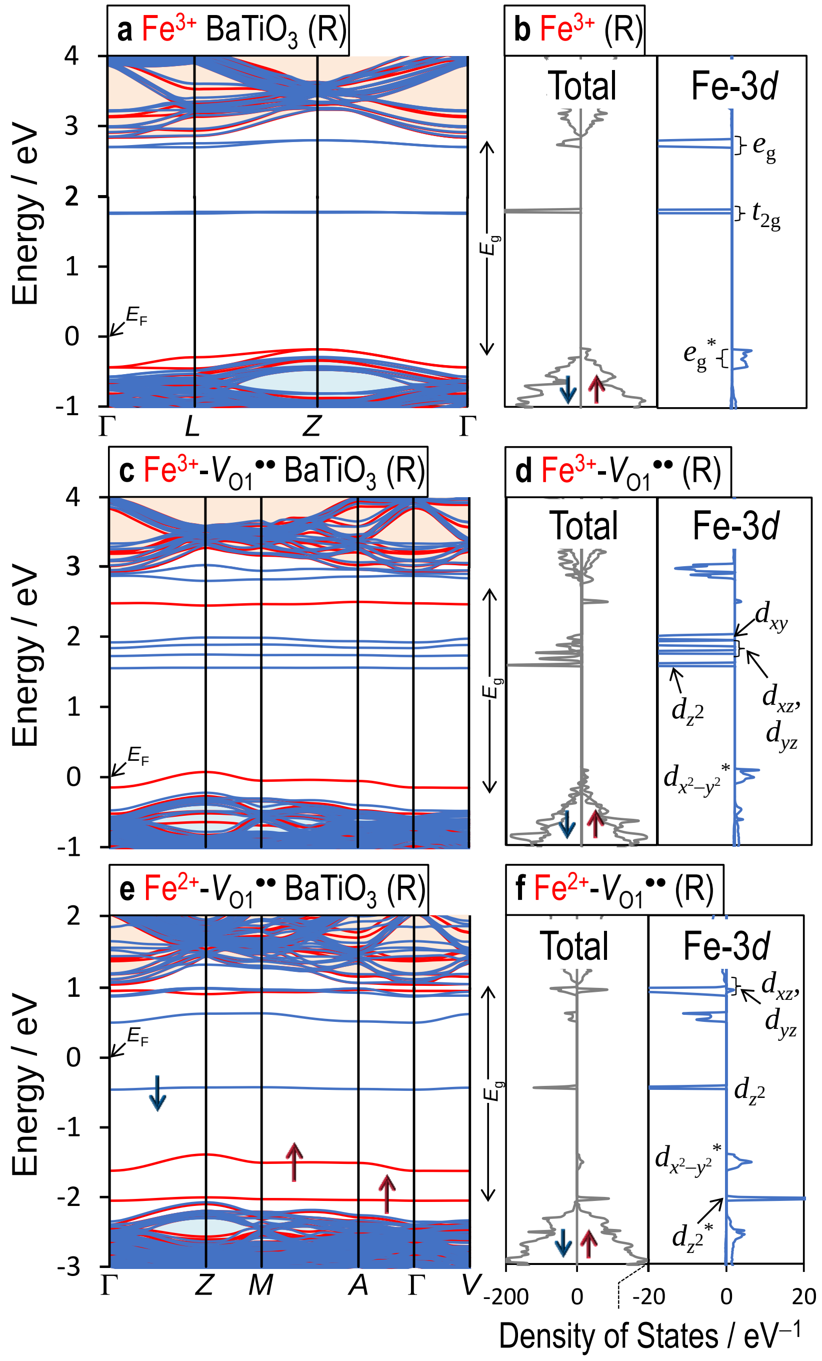

Supplementary Figure 7| Electronic structures of rhombohedral (R) Fe-BaTiO₃ cells. The cells are the supercell of 3 × 3 × 3 created from the optimized (primitive) $BaTiO_3$ structure with $R3m$ symmetry. For generating each the defective cell, we removed one oxygen atom on the nearest-neighbour site with Fe in $P1$ symmetry, i.e., created $V_{O1}^{\bullet\bullet}$, and then raised the symmetry as high as possible. The composition and the space group are as follows: **a, b** $Ba_{27}Ti_{26}FeO_{81}$ (Fe^{3+}, $Z = 3$, space group: $R3m$), **c, d** $Ba_{27}Ti_{26}FeO_{80}$ (Fe^{3+}-$V_{O1}^{\bullet\bullet}$, $Z = 2$, space group: Cm), **e, f** $Ba_{27}Ti_{26}FeO_{80}$ (Fe^{2+}-$V_{O1}^{\bullet\bullet}$, $Z = 2$, space group: Cm). The details of the calculations are the same as those described in **Method**. The overall features are similar to those of the tetragonal (T) cells (Fig. 4)

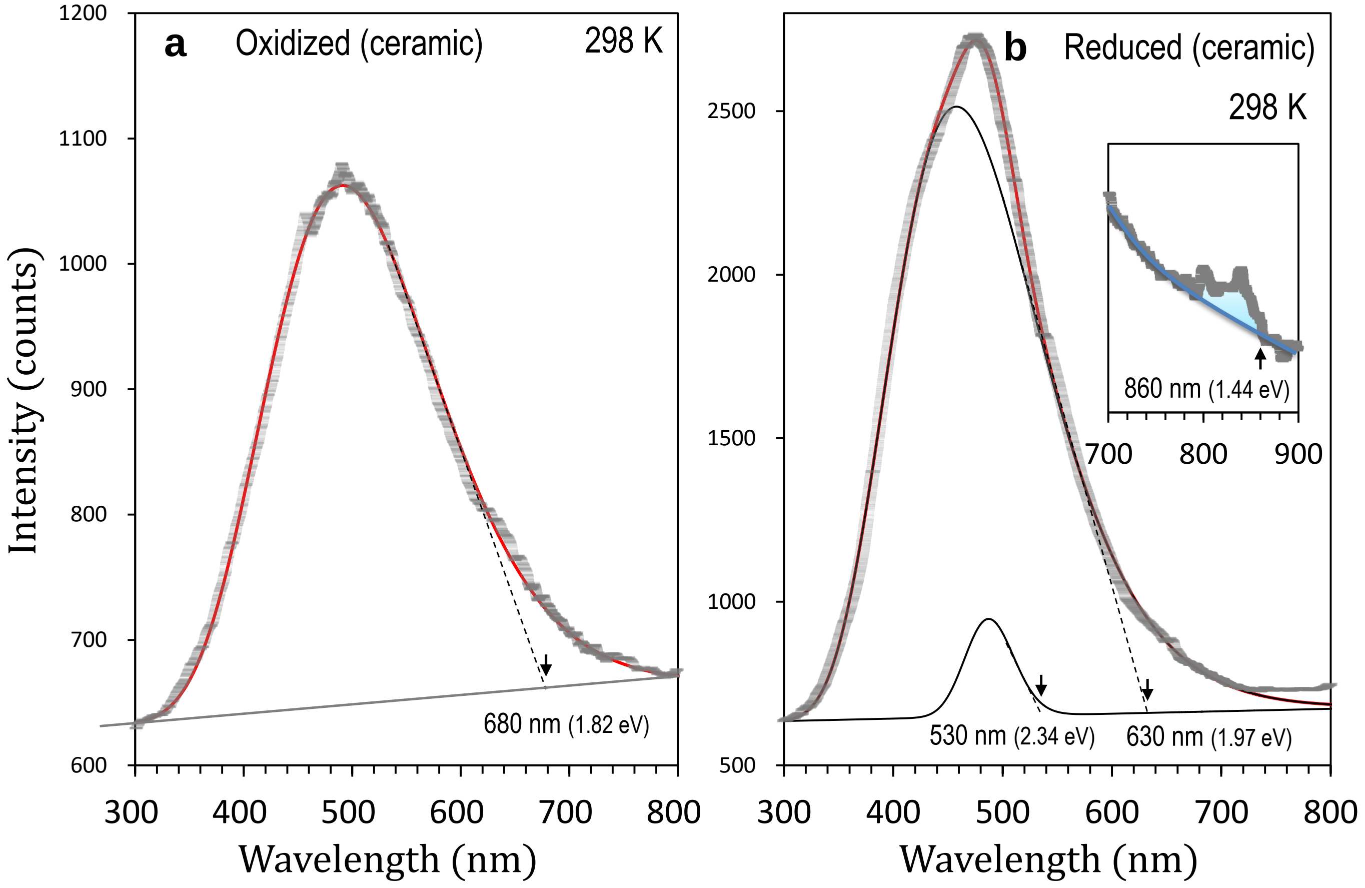

Supplementary Figure 8| Cathode-luminescence (CL) spectra of Fe-BaTiO$_3$ ceramics. The CL data (grey crossbar) were collected at 298 K for BaTiO$_3$ ceramics with an Fe concentration of 1 % (98 % relative density); the reduced sample was annealed at $Po_2^{900\,°C} = 1.0 \times 10^{-24}$ atm for 24 h and the oxidized one at $Po_2^{1{,}200\,°C} = 0.2$ atm (in air) for 24 h, where the superscript denotes the annealing temperature. The CL profile of the oxidized ceramic can be traced by a single log-normal function (red curve with $H = 418.1$, $\lambda_0 = 143.2$ nm, $w = 512.5$ nm, and $\rho = 1.31$). In contrast, that of the reduced ceramic has an apparent shoulder at ≈ 420 nm and could not be fitted by a single function. The CL data were well reproduced by a superposition (red curve) of two log-normal functions (black lines): the parameters of the large peak are $H = 1867$, $\lambda_0 = 208.9$ nm, $w = 446.5$ nm, and $\rho = 1.38$, and those of the small peak are $H = 298$, $\lambda_0 = 81.7$ nm, $w = 142.6$ nm, and $\rho = 1.07$. The oxidized ceramic displays an edge energy of ≈ 1.8 eV, while the reduced ceramic exhibits those of ≈ 1.9 eV and ≈ 2.3 eV. The reduced ceramic has an additional peak in the λ range of 780-860 nm; the edge energy is roughly estimated to be ≈ 1.4 eV (the blue line indicates background)

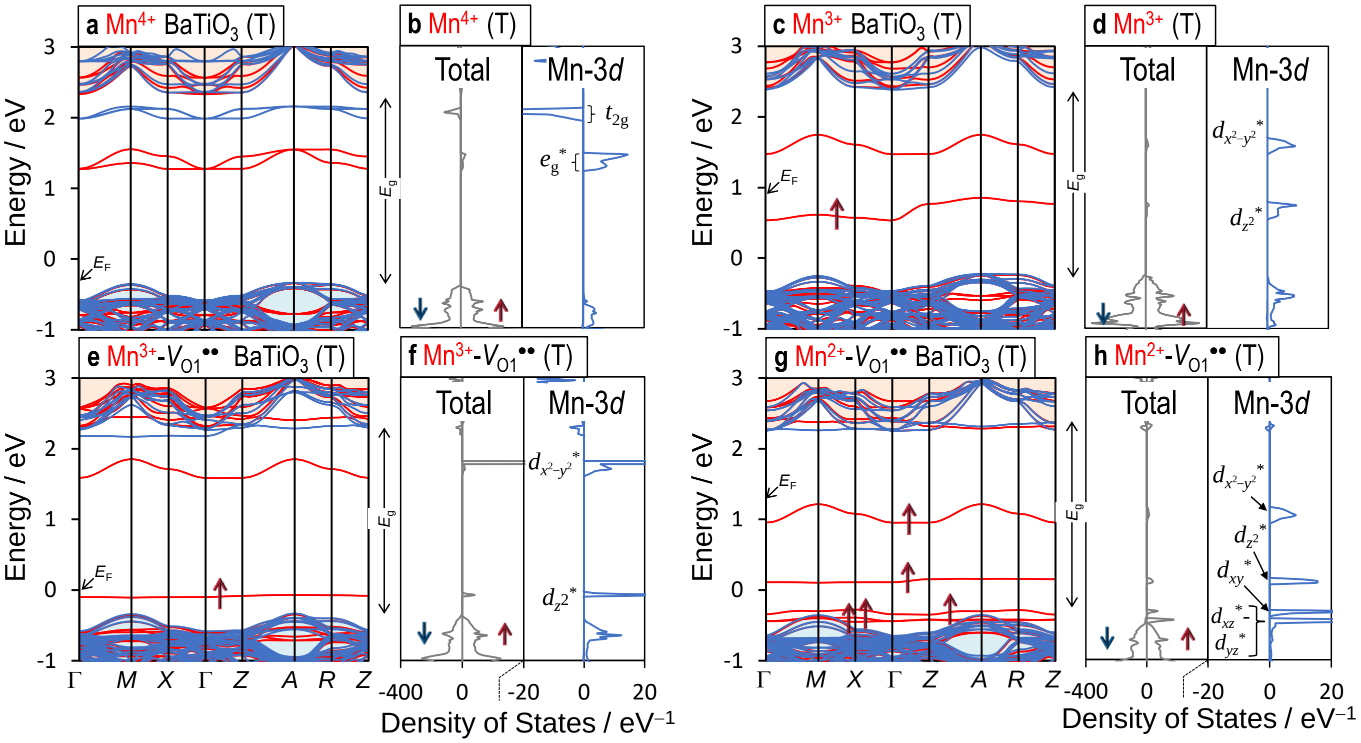

Supplementary Figure 9| Electronic structures of tetragonal (T) Mn-BaTiO₃ cells. Band structures and total (partial) density of states are shown for **a, b** $Ba_{27}Ti_{26}MnO_{81}$ (Mn^{4+}), **c, d** $Ba_{27}Ti_{26}MnO_{81}$ (Mn^{3+}), **e, f** $Ba_{27}Ti_{26}MnO_{80}$ (Mn^{3+}-$V_{O1}^{\bullet\bullet}$), and **g, h** $Ba_{27}Ti_{26}MnO_{80}$ (Mn^{2+}-$V_{O1}^{\bullet\bullet}$), where $V_{O1}^{\bullet\bullet}$ is the oxygen vacancy on the O1 site. The details of the calculations are the same as those described in **Method**.

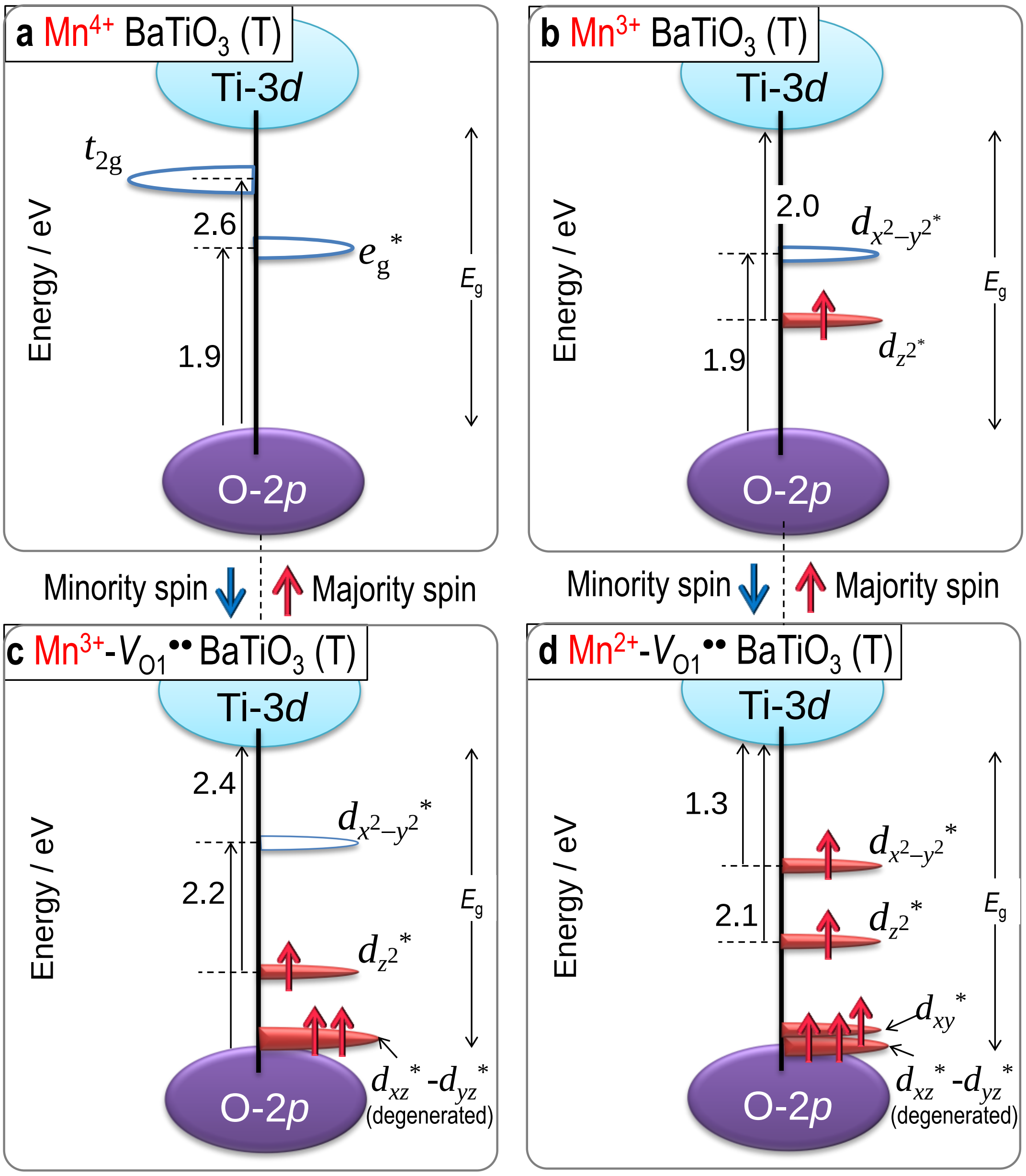

Supplementary Figure 10| Schematics of the gap states for tetragonal (T) Mn-doped BaTiO₃. The electronic structures of the **a** $Ba_{27}Ti_{26}MnO_{81}$ (Mn^{4+}), **b** $Ba_{27}Ti_{26}MnO_{81}$ (Mn^{3+}), **c** $Ba_{27}Ti_{26}MnO_{80}$ (Mn^{3+}-$V_{O1}^{\bullet\bullet}$), and **d** $Ba_{27}Ti_{26}MnO_{80}$ (Mn^{2+}-$V_{O1}^{\bullet\bullet}$) cells are shown, where $V_{O1}^{\bullet\bullet}$ denotes the oxygen vacancy on the O1 site. The Mn^{4+} cell **a** has the empty gap states of t_{2g} (↓) at a depth of 2.6 eV and of e_g^* (↑) at a depth of 1.9 eV from the valence band maximum (VBM). In the **b** Mn^{3+} and **c** Mn^{3+}-$V_O^{\bullet\bullet}$ cells, the unoccupied states of Mn-3d_{x2-y2}^* (↑) and the filled state of Mn-3d_{z2}^* (↑) appear. The Mn^{3+}-$V_O^{\bullet\bullet}$ cell contains the filled Mn-3d_{xz}^*-d_{yz}^* states (↑) in the vicinity of the VBM. The Mn^{2+}-$V_{O1}^{\bullet\bullet}$ involves the occupied Mn-3d derived states (↑): especially, the 3d_{x2-y2}^* state is positioned at a depth of 1.3 eV from the conduction band minimum (CBM) and thereby acts as an electron donor under visible-light illumination. This state can activate the first PV onset. Considering also that the Mn^{3+} cell provides the acceptor state derived from Mn-3d_{x2-y2}^* (↑) at 1.9 eV from the VBM, we expect that Mn-$BaTiO_3$ in the mixed state of Mn^{3+} and Mn^{2+} exhibits a robust PV response as a result of electron-hole pair generation at above $h\nu$ of 1.9 eV.

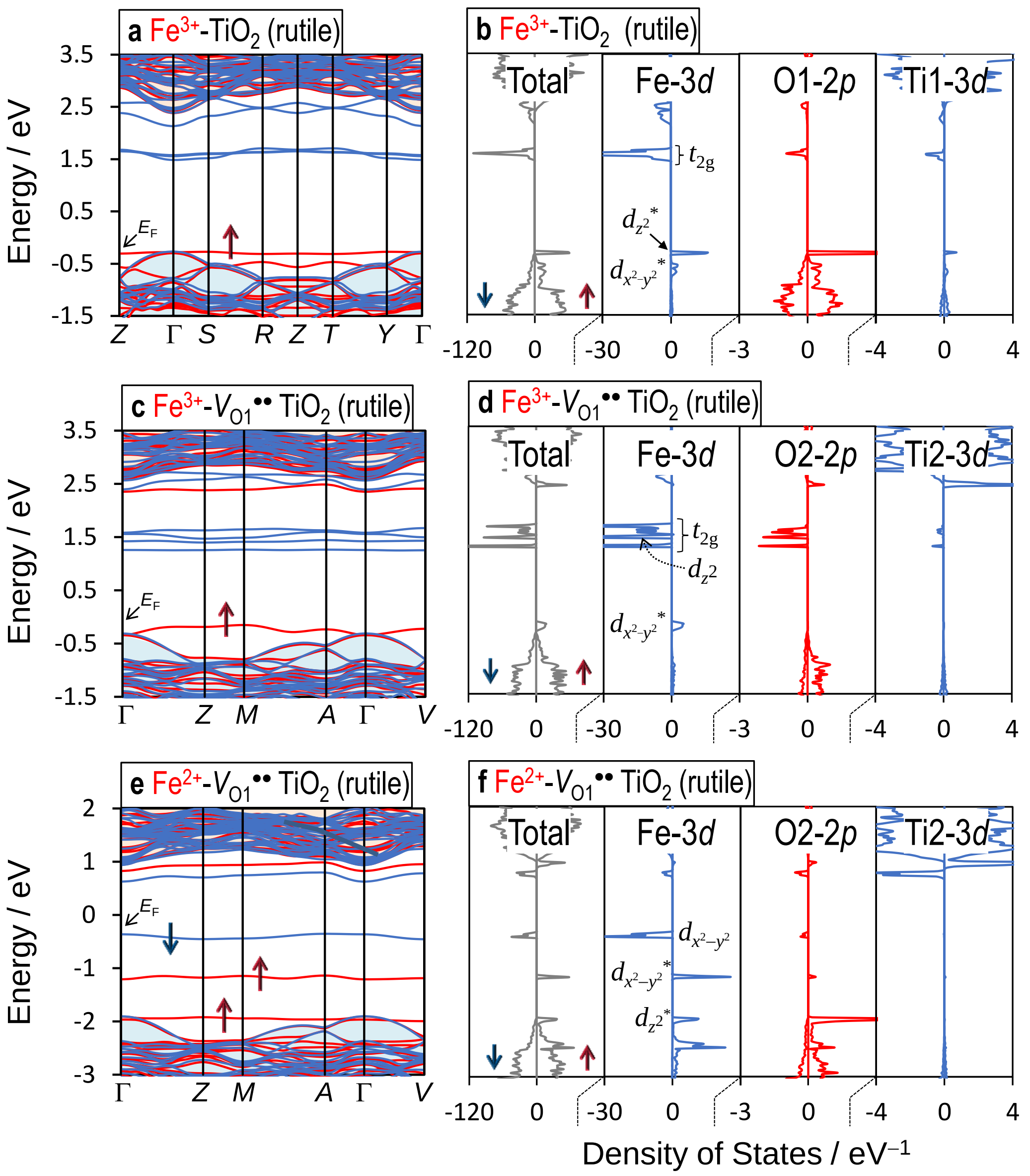

Supplementary Figure 11| Electronic structures of rutile Fe-TiO2 cells. The band structures and the total (partial) density of states are shown for the **a, b** Fe^{3+}, **c, d** Fe^{3+}-$V_{O1}^{\bullet\bullet}$, and **e, f** Fe^{2+}-$V_{O1}^{\bullet\bullet}$ cells, where $V_{O1}^{\bullet\bullet}$ is the oxygen vacancy on the nearest-neighbour oxygen site with Fe ions. The composition and the crystal symmetry are as follows: the Fe^{3+} cell with $Ti_{15}FeO_{32}$ ($Z = 2$) in space group *Cmmm*, the Fe^{3+}-$V_{O1}^{\bullet\bullet}$ cell with $Ti_{15}FeO_{31}$ ($Z = 2$) in *Cm*, and the Fe^{2+}-$V_{O1}^{\bullet\bullet}$ cell with $Ti_{15}FeO_{31}$ ($Z = 2$) in *Cm*. From the optimized structure of rutile TiO_2 ($Z = 2$) in space group $P4_2/mnm$, we created the supercell of 2 × 2 × 2 with the composition of $Ti_{16}O_{32}$. In *P*1 symmetry, one Ti atom was replaced by Fe atom and then the symmetry was transformed to space group *Cmmm* for the Fe^{3+} cell. We also created the defective cells of Fe^{3+}-$V_{O1}^{\bullet\bullet}$ and Fe^{2+}-$V_{O1}^{\bullet\bullet}$ with the composition $Ti_{15}FeO_{31}$ ($Z = 2$, space group *Cm*). All the cells were structurally optimized with a plane-wave cut-off energy of 520 eV, an on-site Coulomb interaction parameter of U–J of 2 eV for Fe-3*d*. The electronic structures were calculated for the optimized cells with an additional U–J parameter of 8 eV for Ti-3*d*. We employed the Γ-centred 3 × 3 × 3 *k*-point mesh for the structural optimizations and the 5 × 5 × 5 *k*-point mesh for the density-of-states and the band structure calculations.

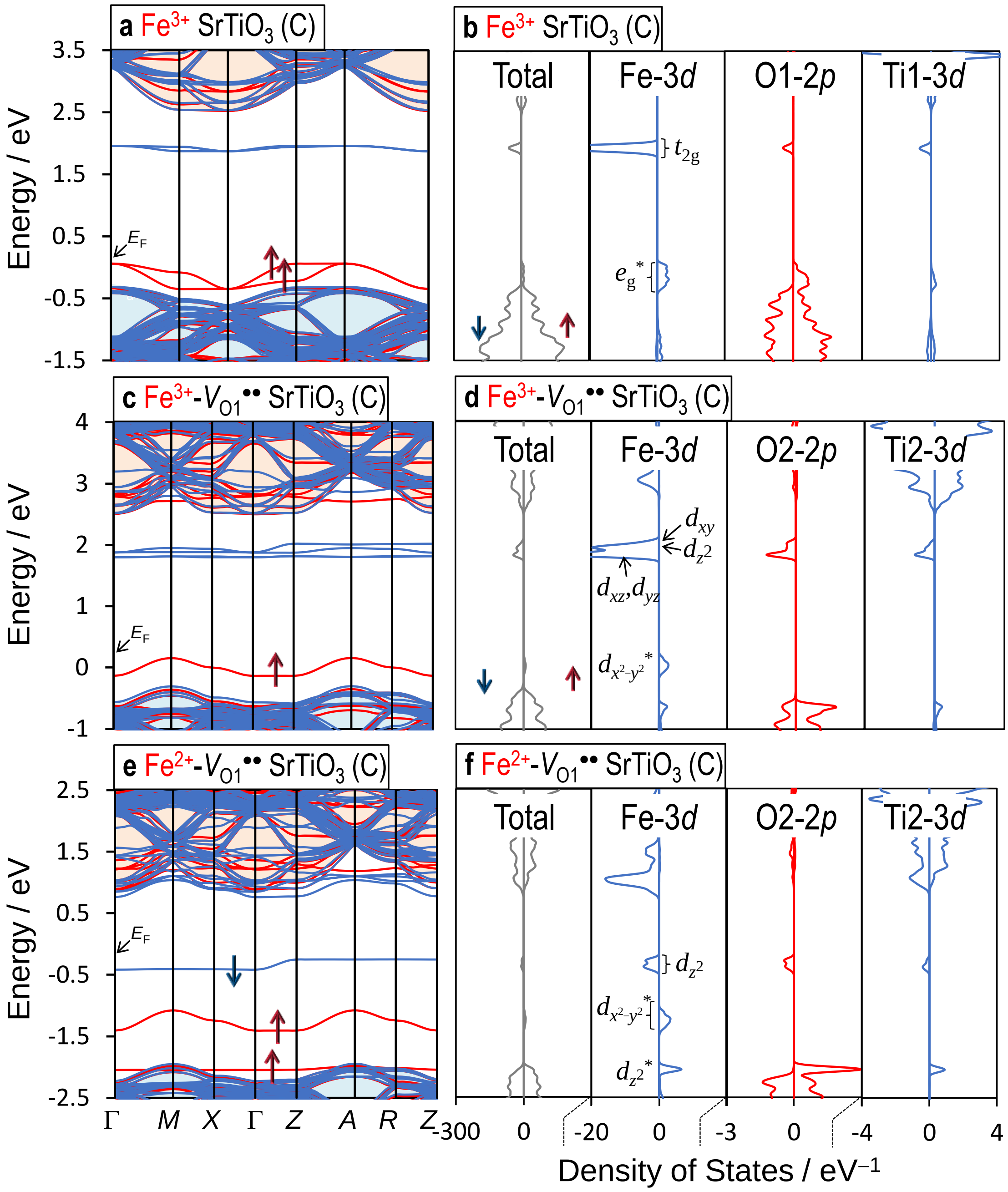

Supplementary Figure 12| Electronic structures of cubic (C) Fe-SrTiO3 cells. The band structures and the total (partial) density of states are shown for **a, b** the $Sr_{27}Ti_{26}FeO_{81}$ (Fe^{3+}) cell in space group $Pm\bar{3}m$, **c, d** the $Sr_{27}Ti_{26}FeO_{80}$ (Fe^{3+}-$V_O^{\bullet\bullet}$) cell in $P4mm$, and **e, f** the $Sr_{27}Ti_{26}FeO_{80}$ (Fe^{2+}-$V_O^{\bullet\bullet}$) cell in $P4mm$, where $V_{O1}^{\bullet\bullet}$ is the oxygen vacancy on the nearest-neighbour site with Fe). The details of the calculations are the same as those described in **Method**.

Supplementary Table 1| Bulk photovoltaic tensor elements. Non-zero components of the bulk photovoltaic (PV) tensor elements (β_{31} and β_{33}) at photon energy (*hv*) of 3.1 eV (405 nm). It is confirmed that β_{15} is at least one order of magnitude smaller than β_{33} for both the samples.

Single crystal samples	Bulk photovoltaic tensor elements (× 10^{-9} V^{-1})	
	β_{31}	β_{33}
Fe-doped $BaTiO_3$ (reduced)	−55.4	−32.5
Fe-doped $BaTiO_3$ (oxidized)	−2.86	−1.77
Mn-doped $BaTiO_3$ (oxidized)[1]	−7.86	−1.57
Non-doped $BaTiO_3$ (oxidized) [1]	−0.12	−0.004

Supplementary Note 1. Defect Concentration Calculations

The defect concentrations of Fe^{2+} (Fe_{Ti}''), Fe^{3+} (Fe_{Ti}'), and Fe^{4+} ($Fe_{Ti}^{\times}$) on the Ti site, oxygen vacancy ($V_O^{\bullet\bullet}$), and thermally activated electron (e') and hole ($h^{\bullet}$) were calculated by solving nonlinear equations based on the defect chemistry, where the reported values of the mass-action constants and the enthalpies etc.[4–8] were employed. The singly charged oxygen vacancy ($V_O^{\bullet}$) has been reported in the $SrTiO_3$ system, while the concentration of $V_O^{\bullet}$, $[V_O^{\bullet}]$, in Fe-doped $SrTiO_3$ is several orders of magnitude smaller than $[V_O^{\bullet\bullet}]$[8], where square brackets denote volumetric concentration, i.e., number of defects per cubic centimeter. As far as we are aware, the major contribution of $V_O^{\bullet}$ has not been reported for $BaTiO_3$[2,9]. We therefore consider only $V_O^{\bullet\bullet}$ as an oxygen vacancy throughout this study.

It is reasonable to consider that the concentration of Fe (0.3%) remains constant not only in the annealing at a temperature (T) of 900 °C but also after the quenching to room temperature, as assumed for transition-metal doped $BaTiO_3$ in the literature[8,10–14]. At high temperatures, such as 900 °C, the kinetics of oxygen exchange between perovskite oxides and the surrounding gas phase is relatively fast[12,15]. We then think that the oxygen activity inside the samples is in an equilibrium state.

As previously reported, Fe at the Ti^{4+} site has the valence states of Fe^{4+}, Fe^{3+}, and Fe^{2+} [7,8,10]. The total concentration of Fe remains unchanged (m_{Fe} denotes dosage of Fe):

$$m_{Fe} = [Fe_{Ti}''] + [Fe_{Ti}'] + [Fe_{Ti}^{\times}]. \quad (1)$$

The electroneutrality condition regarding all charged defects is expressed as

$$2[Fe_{Ti}''] + [Fe_{Ti}'] + n = 2[V_O^{\bullet\bullet}] + p, \quad (2)$$

where n and p are e' and $h^{\bullet}$ concentrations, respectively. The equilibrium of thermally activated e'-$h^{\bullet}$ generation through the band gap can be written as

$$null \leftrightarrow e' + h^{\bullet}. \quad (3)$$

The product of n and p coincides with the intrinsic constant of $K_I(T)$ as

$$K_I(T) = n \cdot p = N_C(T) \cdot N_V(T) \exp\{-[E_g(0K) - \beta T]/kT\} \quad (4)$$

$N_C(T) = 4.1 \times 10^{16}$ cm^{-3}(T K^{-1})$^{2/3}$ and $N_V(T) = 3.5 \times 10^{16}$ cm^{-3}(T K^{-1})$^{2/3}$ are the effective densities of states in the valence band and conduction band, respectively[4] and k denotes the Boltzmann constant. We employ the following constants representing the band gap E_g(0 K) [5,6] and its temperature coefficient β: E_g(0 K) = 3.5 eV and $\beta = 6.0 \times 10^{-4}$ eV K^{-1}. The formation of $V_O^{\bullet\bullet}$ by the oxygen kinetics is written as

$$O_O^{\times} \leftrightarrow V_O^{\bullet\bullet} + 1/2O_2 + 2e', \quad (5)$$

and the mass-action constant of this reduction reaction (K_{Red}) is denoted as

$$K_{Red} = [V_O^{\bullet\bullet}]P_{O_2}^{1/2}n^2 = K_{Red}^{\circ} \exp(-\Delta H_{Red}/kT), \quad (6)$$

with $K_{Red}^{\circ} = 1.7 \times 10^{72}$ cm^{-9} atm$^{1/2}$ [4]. In a similar manner as E_g, we adopt a T-dependent $\Delta H_{Red} = \Delta H_{Red}$ (0 K) $- \gamma T$ with ΔH_{Red} (0 K) = 6.0 eV and $\gamma = 1.0 \times 10^{-4}$ eV K^{-1}, which is slightly modified from the reported values in the literature[4].

The ionization reactions of Fe on the Ti^{4+} site can be described by the generations of e' or $h^{\bullet}$, while those expressed by using e' and $h^{\bullet}$ are exactly identical from the viewpoint of the defect chemistry[2,16–21]. Here, we use the expressions based on the $h^{\bullet}$ generation. Fe ions can be ionized negatively by producing $h^{\bullet}$, and the ionizations of Fe ions are expressed in terms of the $h^{\bullet}$ generation reactions:

$Fe_{Ti}' \leftrightarrow Fe_{Ti}'' + h^{\bullet}$, ($K_{h1}$, ΔH_{h1}), (7)

$Fe_{Ti}^{\times} \leftrightarrow Fe_{Ti}' + h^{\bullet}$, ($K_{h2}$, ΔH_{h2}), (8)

where their respective equilibrium constants (K_{h1} and K_{h2}) and enthalpies (ΔH_{h1} and ΔH_{h2}) are written as

$K_{h1} = p[Fe_{Ti}'']/[Fe_{Ti}'] = K_{h1}^{\circ} \exp(-\Delta H_{h1}/kT)$ (9)

$K_{h2} = p[Fe_{Ti}']/[Fe_{Ti}^{\times}] = K_{h2}^{\circ} \exp(-\Delta H_{h2}/kT)$ (10)

where $K_{h1}^{\circ} = N_V(T)$ and $K_{h2}^{\circ} = N_V(T) = 3.5 \times 10^{16}$ $cm^{-3}(T\ K^{-1})^{2/3}$ are their respective equilibrium constants[4]. Because E_g is temperature dependent, the ionization enthalpies ΔH_{h1} and ΔH_{h2} also depend on temperature as follows: $\Delta H_{h1} = \Delta H_{h1}$ (0 K) $- \beta T$ [ΔH_{h1} (0 K) = 2.87 eV] and $\Delta H_{h2} = \Delta H_{h2}$ (0 K) $- \beta T$ [ΔH_{h2} (0 K) = 1.27 eV], which are derived from the reported parameters[4,8,10].

When the temperature is lowered, the long-range ionic motion fails to reach its equilibrium. At low temperatures below 400 °C [22], we can assume that [$V_O^{\bullet\bullet}$] in the high-temperature equilibrium state is frozen-in because the oxygen exchange kinetics is extremely slow in this quenched state[8,12–14,23]. In contrast that the reduction reaction of Eq. (5) is frozen, the internal electronic reactions of the bandgap excitation (3) and the electron-trapping reactions of Fe ions [(7) and (8)] are still active even at low temperatures[8,12–14,23]. We therefore examined the defect concentrations as follows: (1) we first calculated the defect concentrations in the high-temperature equilibrium state at 900 °C as a function of oxygen partial pressure $Po_2^{900\ °C}$, (2) under the fixed value of [$V_O^{\bullet\bullet}$] at each $Po_2^{900\ °C}$, other all concentrations in the quenched state were calculated as a function of T.

Supplementary References

1. Inoue, R. *et al.* Giant photovoltaic effect of ferroelectric domain walls in perovskite single crystals. *Sci. Rep.* **5**, 14741 (2015).
2. Chan, N.-H., Sharma, R. K. & Smyth, D. M. Nonstoichiometry in Undoped $BaTiO_3$. *J. Am. Ceram. Soc.* **64**, 556–562 (1981).
3. Possenriede, E., Jacobs, P. & Schirmer, O. F. Paramagnetic defects in $BaTiO_3$ and their role in light-induced charge transport. I. ESR studies. *J. Phys. Condens. Matter* **4**, 4719–4742 (1992).
4. Moos, R. & Hardtl, K. H. Defect Chemistry of Donor-Doped and Undoped Strontium Titanate Ceramics between 1000° and 1400°C. *J. Am. Ceram. Soc.* **80**, 2549–2562 (2005).
5. Schrader, M., Mienert, D., Oh, T.-S., Yoo, H.-I. & Becker, K. D. An optical, EPR and electrical conductivity study of blue barium titanate, $BaTiO_{3-\delta}$. *Solid State Sci.* **10**, 768–775 (2008).
6. Baer, W. S. Interband faraday rotation in some perovskite oxides and rutile. *J. Phys. Chem. Solids* **28**, 677–687 (1967).
7. Hagemann, H.-J. & Hennings, D. Reversible Weight Change of Acceptor-Doped $BaTiO_3$. *J. Am. Ceram. Soc.* **64**, 590–594 (1981).
8. Saraf, S., Markovich, M. & Rothschild, A. Defect chemistry of pn junctions in complex oxides. *Phys. Rev. B* **82**, 245208 (2010).
9. Hagemann, H.-J. & Ihrig, H. Valence change and phase stability of 3*d*-doped $BaTiO_3$ annealed in oxygen and hydrogen. *Phys. Rev. B* **20**, 3871–3878 (1979).
10. Wechsler, B. A. & Klein, M. B. Thermodynamic point defect model of barium titanate and application to the photorefractive effect. *J. Opt. Soc. Am. B* **5**, 1711 (1988).
11. Lambeck, P. V. & Jonker, G. H. The nature of domain stabilization in ferroelectric perovskites. *J. Phys. Chem. Solids* **47**, 453–461 (1986).
12. Maier, J. High temperature versus low temperature defect chemistry. *Solid State Ionics* **173**, 1–8 (2004).
13. Yoo, H.-I., Oh, T.-S., Kwon, H.-S., Shin, D.-K. & Lee, J.-S. Electrical conductivity-defect structure correlation of variable-valence and fixed-valence acceptor-doped $BaTiO_3$ in quenched state. *Phys. Chem. Chem. Phys.* **11**, 3115–26 (2009).
14. Yoon, S.-H., Randall, C. A. & Hur, K.-H. Difference between resistance degradation of fixed valence acceptor (Mg) and variable valence acceptor (Mn)-doped $BaTiO_3$ ceramics. *J. Appl. Phys.* **108**, 064101 (2010).
15. Yoo, H. I., Song, C. R., Lee, Y. S. & Lee, D. K. Surface reaction kinetics in oxygen nonstoichiometry re-equilibration of $BaTiO_{3-\delta}$. *Solid State Ionics* **160**, 381–387 (2003).
16. Raymond, M. V. & Smyth, D. M. Defects and charge transport in perovskite ferroelectrics. *J. Phys. Chem. Solids* **57**, 1507–1511 (1996).
17. Zhang, X. W., Han, Y. H., Lal, M. & Smyth, D. M. Defect Chemistry of $BaTiO_3$ with Additions of $CaTiO_3$. *J. Am. Ceram. Soc.* **70**, 100–103 (1987).
18. Chan, N.-H., Sharma, R. K. & Smyth, D. M. Nonstoichiometry in Acceptor-Doped $BaTiO_3$. *J. Am. Ceram. Soc.* **65**, 167–170 (1982).
19. Han, Y. H., Appleby, J. B. & Smyth, D. M. Calcium as an Acceptor Impurity in $BaTiO_3$. *J. Am.*

Ceram. Soc. **70**, 96–100 (1987).

20. Chan, N.-H. & Smyth, D. M. Defect Chemistry of Donor-Doped $BaTiO_3$. *J. Am. Ceram. Soc.* **67**, 285–288 (2006).
21. Chan, H. M., Harmer, M. R. & Smyth, D. M. Compensating Defects in Highly Donor-Doped $BaTiO_3$. *J. Am. Ceram. Soc.* **69**, 507–510 (1986).
22. Denk, I., Münch, W. & Maier, J. Partial Conductivities in $SrTiO_3$: Bulk Polarization Experiments, Oxygen Concentration Cell Measurements, and Defect-Chemical Modeling. *J. Am. Ceram. Soc.* **78**, 3265–3272 (1995).
23. Merkle, R. & Maier, J. How Is Oxygen Incorporated into Oxides? A Comprehensive Kinetic Study of a Simple Solid-State Reaction with $SrTiO_3$ as a Model Material. *Angew. Chemie Int. Ed.* **47**, 3874–3894 (2008).
